# The Blues; Causes and Cures

**Published:** 1911-05

**Authors:** 


**The Blues; Causes and Cures.** By **Albert Abrams,** A.M., M.D., Late Professor of Pathology and Director of the Medical Clinic, Cooper Medical College, San Francisco, Cal. Fourth edition. 8vo. 304 pages. Illustrated. E. B. Treat & Co., New York. (Cloth, $1.50.)

This work which treats of the causes and cure of neurasthenia was first published in 1904. The second edition appeared in 1905, the third in 1908 and the present (fourth) in December, 1909. As the previous editions have been reviewed in the Journal, it should be mentioned that new data has been added to the work. In note 20 as an appendix to the work, Abrams describes his method of augmenting the tone of the splanchnic circulation. He believes that intraabdominal venous congestion is present in neurasthenia. The treatment advised and fully described is either concussion or sinusoidalisation of the spine. The first is considered more efficacious.

The work is fully illustrated with cuts demonstrating the various modes of massage advocated by the author. L. K.

